# The Effect of Temperature and Moisture Content of Stored Rapeseed on the Phytosterol Degradation Rate

**DOI:** 10.1007/s11746-012-2064-4

**Published:** 2012-04-24

**Authors:** Marzena Gawrysiak-Witulska, Magdalena Rudzińska, Jolanta Wawrzyniak, Aleksander Siger

**Affiliations:** 1 Institute of Food Technology of Plant Origin, Poznan University of Life Sciences, Wojska Polskiego 31, 60-637 Poznan, Poland; 2 Department of Food Biochemistry and Analysis, Poznan University of Life Sciences, Wojska Polskiego 31, 60-637 Poznan, Poland

**Keywords:** Rapeseed, Phytosterols, Degradation of phytosterols, Postharvest

## Abstract

The effect of temperature (25 or 35 °C) and moisture content (10, 12.5, 15.5 %) on rapeseed phytosterol degradation was examined for 18 days. Statistical analysis showed that temperature, moisture and time of storage have a significant effect on phytosterol degradation. After 18 days of seed storage at a temperature of 25 and 30 °C losses of these compounds amounted to 11 and 13 % in seeds with moisture contents of 10, 12 and 16 % in seeds with a moisture content of 12.5 %, while they were 24 and 58 % in seeds with a moisture content of 15.5 %. Among all the identified sterols the greatest degradation rate was observed for stigmasterol and brassicasterol. Losses of stigmasterol and brassicasterol during storage of seeds with a 12.5 % moisture content at a temperature of 30 °C were 17 and 28 %, respectively, while in seeds with a moisture content of 15.5 % these losses increased to 73 and 63 %.

## Introduction

Rapeseed production in Europe in the last 10 years has increased two-fold from 11.7 to 23 million tonnes, of which 2 million tonnes of seeds are produced in Poland (http://www.faostat.fao.org). The considerable interest in rapeseed observed worldwide results from the fact that this crop contains up to 49 % fat and over 20 % protein, which constitutes a valuable raw material for the fat and oil industry, as well as the animal feed industry [1]. Like other seeds, rapeseed is physiologically active and can be affected by moisture content, temperature and access to oxygen. Immediately after harvest, depending on the weather conditions, the moisture content in seeds may reach 18 %. For their safe storage immediately after harvest, seeds are dried and cooled. The recommended storage moisture content of seeds ranges from 7 to 10 %. In Poland, it is recommended to dry seeds to be stored over prolonged periods of time to a moisture content of 7 % [2]. A maximum moisture content of 10 % is allowed in Canada for seeds to be sold as straight grade (dry) [3], while in Australia a maximum concentration of moisture may be 8 % at seed delivery. However, drying and cooling of rapeseeds alone do not guarantee seed storage to proceed without quality losses. It is common that storage bins undergo heating and cooling, which could alter seed quality. In such a case, a slow movement of air occurs, which is accompanied by migration of moisture, whereby warmer layers are slightly dried, and cooler layers are re-wetted. In the wetted seed layers, biochemical processes are activated, leading to the occurrence of self-heating and the reduction of seed viability and quality including the development of metabolites such as free fatty acids [4, 5]. Degradation of biologically active compounds may take place during self-heating.

Sterols, tocopherols and phenolic compounds found in rapeseed are bioactive components exhibiting antioxidant action. Sterols and sterol esters of fatty acids predominate among the non-acylglycerol lipids of vegetable oils. The total sterols (sum of esterified and non-esterified sterols) generally account for 0.2–1.0 % of the total lipids for most vegetable oils. Biological properties of sterols, particularly the capacity to reduce blood cholesterol level, have resulted in considerable interest in these compounds on the part of the pharmaceutical and food industries [6]. They inhibit absorption of dietary cholesterol in the large intestine, since they compete with this compound for space in emulsion micelles transporting systemic lipids in the blood to the liver. This action has a beneficial effect on the entire organism, since it promotes maintenance of full activity and health of the heart, prostate, liver and the immune system [7].

Phytosterol composition in individual oil crops is species characteristic, which may be used in the identification of oil produced from these oil crops. Thus, potential oil adulterations may be detected on the basis of the qualitative and quantitative composition of the sterols in vegetable oils. [8]. Rapeseed oil contains 0.5–1.1 % phytosterols, of which the total phytosterol fractions are comprised of 45–60 % sitosterol, 25–39 % campesterol, 5–13 % brassicasterol, 3–7 % avenasterol and <1 % stigmasterol [9]. The total content of phytosterols in rapeseed oil (4.6–9.0 mg/g) is approximately twice the amount of that in sunflower (2.1–4.5 mg/g) or soybean (2.3–4.7 mg/g) oils [10].

The content of biologically active compounds in seeds to be used in oil production is, to a considerable degree, dependent on preservation conditions and seed storage conditions after harvest. Gawrysiak-Witulska et al. [5] evaluated changes in tocopherol contents in stored rapeseed. They found that during storage of seeds with a moisture content of 10–15.5 % at a temperature of 25 and 30 °C, losses of tocochromanols and plastochromanols after 18 days amounted to 3.7–14.4 % and 4–24 %, respectively. As there is no available literature on the effect of moisture content and storage temperature on phytosterols content in rapeseed, the objective of this research was to determine the loss of phytosterols during adverse storage conditions of intact seeds.

## Materials and methods

### Materials

The degradation rate of phytosterols in rapeseed during storage under adverse conditions was assessed for seeds of *Brassica napus* L. cv. *Californium*. Seeds were harvested at the Złotniki Experimental Station, belonging to the Poznan University of Life Sciences. Rapeseeds were wetted until the assumed storage conditions were reached according to our previous report [5], after conditioning, the moisture content of rapeseed was 10, 12.5 or 15.5 %.

### Seed Storage

The moisture adjusted seeds were stored in a specially designed thermo-hygrostatic chamber according to our previous method [5]. In this way, the rapeseed inter-seed spaces were subjected to relative humidity (75, 85 and 91 % for NaCl, KCl and BaCl_2_, respectively) corresponding to the assumed equilibrium moisture content of rapeseed of 10, 12.5 and 15.5 %. Relative humidity and temperature were measured as described previously [5]. Two experiments were conducted. Wetted rapeseeds (with a moisture content of 10, 12.5 and 15.5 %) were stored for 18 days at a temperature of 25 and 30 °C. Samples were collected every 6 days during seed storage and stored in sealed plastic containers at −18 °C until analyzed.

### Determination of Seed Moisture, Oil and Sterol Contents

Seed moisture was determined using a moisture balance following our previous method [5]. Oil extraction was completed using the Folch method [11] on 10 g of ground rapeseed and 100 ml chloroform:methanol (2:1 v/v). The solvent was washed with 0.25 volumes of water, vortexed for several seconds and then centrifuged (2,000 rpm) to separate the two phases. The lower chloroform layer was collected and evaporated under vacuum using a Buchi R 215 rotoevaporator.

A saponification method of the AOCS [12] was used to determine the sterol composition. The GC and GC–MS methods of Ciftci et al. [13] were used to identify and quantify the individual sterols. These methods use a DB-35MS capillary column (25 m × 0.20 mm, 0.33 μm J&W Scientific, Folsom, CA) for a GC analysis and a DB-5 capillary column (50 m 9 0.2 mm, 0.32 mm; J&W Scientific, Folsom, CA) for GC–MS. Other conditions used were as reported by Ciftci et al. [13].

### Statistical Analysis

Results are presented as means ± standard deviation from three replicates of each experiment. Significant differences (*p* < 0.05) between mean values were determined by the three factorial analysis of variance (ANOVA) where time was the repeated measure. Interactions between analyzed factors were determined in the ANOVA. Calculations were performed with Statistica 10.0 (StatSoft, Inc., Tulsa, OK) software.

## Results and Discussion

**Fig. 1:**
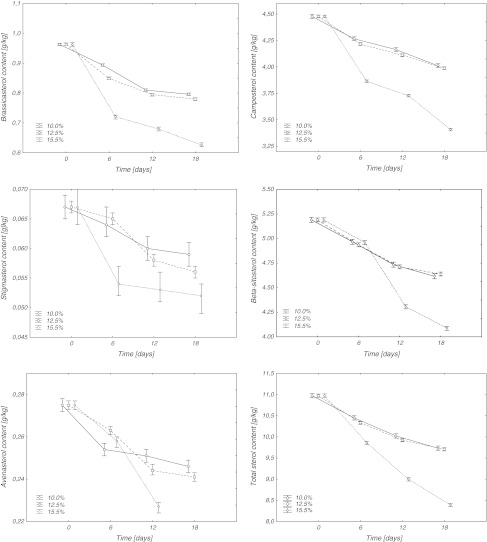
Phytosterol content in rapeseed with different moisture contents stored at 25 °C

**Fig. 2:**
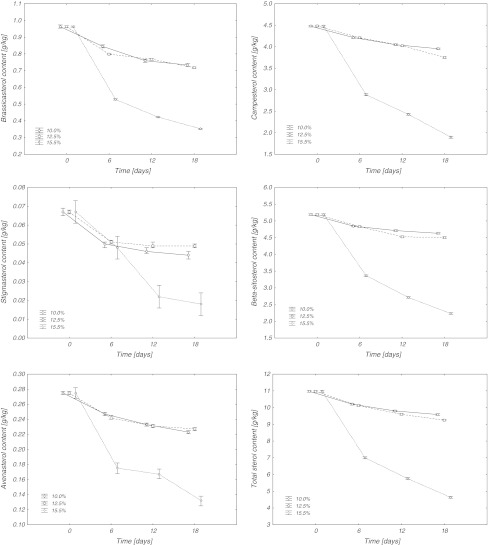
Phytosterol content in rapeseed with different moisture contents stored at 30 °C

The contents of five major plant sterols, i.e. brassicasterol, campesterol, stigmasterol, sitosterol and avenasterol, were determined after saponification of oil extracted from all separated rapeseed samples. Since the sterol fraction contained very few peaks at low intensity (data not shown here), no effort was made to identify 4-monomethylsterols and 4,4′-dimethylsterols. Changes in contents of individual phytosterols and total sterols in rapeseed, stored at 25 and 30 °C, are presented in Figs. 1 and 2. In rapeseed harvested from the field the total content of phytosterols for the analyzed *Brassica napus* cv. *Californium* amounted to 10.97 g/kg fat. The level of these compounds in rapeseed results from cultivar specific traits, moisture content in seeds as well as their harvest and storage conditions [14]. Percentages of the sterol fractions in analyzed seeds were typical of rapeseed oil. The dominant sterol was β-sitosterol (5.19 g/kg), which accounted for 47 % of total contents of sterols and campesterol (4.48 g/kg), accounting for 41 % of the sterol fraction. Brassicasterol (0.96 g/kg), a characteristic sterol of cruciferous plants, in oils extracted from seeds constituted 8.5 % of all sterols. The other sterols were found in much smaller amounts. The content of stigmasterol was 0.07 g/kg, while for avenasterol it was 0.27 g/kg oil, which corresponded to 1 and 2.5 % sterol contents, respectively.

The total content of sterols observed in all samples decreased during storage (Figs. 1, 2). However, the rate of these changes was dependent both on the moisture content of seeds and the temperature during storage. Seeds with moisture contents of 10 and 12.5 % stored at 25 °C, showed an 11–12 % reduction in total sterol contents after 18 days of storage. Seeds stored at 30 °C had slightly higher sterol losses that amounted to 13 and 16 %, respectively. Much greater losses of phytosterols were recorded for seeds with a moisture content of 15.5 %. Storage of such moist seeds at 25 and 30 °C resulted in the loss of sterols amounting to 24 and 58 %, respectively.

The degradation of individual sterols under the described storage conditions was also evaluated (Figs. 1, 2). A comparison of the degradation rate for individual sterols showed that stigmasterol and brassicasterol were degraded much faster than the other identified sterols (Figs. 1, 2). Seeds with a moisture content of 10 %, stored for 18 days at 25 °C, had a 12 % loss in stigmasterol, while at 30 °C the losses were two-fold and amounted to 25 %. An identical storage time for seeds with a moisture content of 12.5 % resulted in 17 and 27 % stigmasterol losses at 25 and 30 °C, respectively. A 23 % loss in stigmasterol was observed in seeds at a 15.5 % moisture content during storage at 25 °C. An increase in storage temperature to 30 °C considerably accelerated the degradation of stigmasterol and increased losses to 73 %. Seeds with a 10 % moisture content after 18 days of storage lost from 17 to 24 % of brassicasterol depending on the temperature. An increase in seed moisture content to 12.5 % caused losses in brassicasterol of 19 and 26 %, whereas for a moisture content of 15.5 %, losses amounted to 35 and 63 % for 25 and 30 °C, respectively. A much greater degradation of stigmasterol and brassicasterol resulted probably due to their chemical structure, which differs from that of the other compounds from that group. Stigmasterol, i.e. stigmasta-5,22-dien-3β-ol, and brassicasterol, i.e. ergosta-5,22-dien-3β-ol, have double bonds in their structure between the 22nd and 23rd atoms of carbon in the lateral chain [15].

Degradation of β-sitosterol, avenasterol and campesterol for the storage conditions was similar. During storage of seeds with moisture contents of 10 and 12.5 % at 25 °C losses of 10–12 % were observed. An increase in temperature for seeds with a 10 % moisture content up to 30 °C did not increase losses of β-sitosterol and campesterol, while avenasterol losses reached 19 %. An increase in storage temperature to 30 °C for seeds with a moisture content of 12.5 % did not increase β-sitosterol losses, but resulted in increased losses of avenasterol and campesterol, which after 18 days of storage amounted to 16–17 %. The highest losses in β-sitosterol, avenasterol and campesterol were recorded after 18 day storage of seeds with a moisture content of 15.5 %. At 25 °C, losses amounted to 21–24 %, while during storage at a temperature of 30 °C they more than doubled to 52–58 %.

Generally, degradation of sterols depends on the structure of the side chain, especially on the number of double bonds. However, in this experiment differences in sterol degradation were observed only in samples stored under the most drastic conditions (15.5 % moisture content, 30 °C, 18 days). Differences between the degradation of sterols in other samples were not statistically significant.

Statistical analysis showed that temperature, moisture and time of storage are significant. All two and three factor interactions were significant (Table 1). Post hoc analyses (Tukey’s tests) showed that, with an increase in moisture content, a significant reduction was observed in sterol contents during storage. For all phytosterols, the greatest decrease during storage was observed at a 15.5 % moisture content. The loss of total phytosterol contents after 12 days of storage at 12 % moisture content and at 30 °C did not differ significantly from that in samples stored for 18 days at a 10 % moisture content and 30 °C. Total phytosterol losses after 6 days of storage at a 15.5 % moisture content and 25 °C were the same as after 12 and 18 day of storage at a 10 % moisture level and 25 and 30 °C, respectively. Similar results were observed for particular phytosterols.

**Table 1:** Three factorial analysis of variance for total sterol content in rapeseed

Effect	Sum of squares (SS)	*df*	Mean of square (MS)	*F* statistic	*p* value
Moisture (*M*)	5.438E + 07	2	2.719E + 07	5,724	<0.0001
Temperature (*T*)	1.635E + 07	1	1.635E + 07	3,442	<0.0001
*M* × *T*	2.050E + 07	2	1.025E + 07	2,158	<0.0001
Time	5.994E + 07	3	1.998E + 07	150,270	<0.0001
Time × *M*	2.103E + 07	6	3.505E + 06	26,364	<0.0001
Time × *T*	5.730E + 06	3	1.910E + 06	14,364	<0.0001
Time × *M* × *T*	7.222E + 06	6	1.203E + 06	9,052	<0.0001
Error	4.787E + 03	36	1.329E + 02		

Results presented in this paper show that the moisture content in rapeseed and the temperature of storage significantly influence the phytosterol degradation. This is in agreement with results obtained in previous studies [14, 16]. According to Rudzinska et al. [14], losses of total sterols after a 1-year storage of seeds with a moisture content of 7 %, dried after harvest with air at a temperature within the range of 60–120 °C, may reach 13 %, while the biggest losses were recorded for stigmasterol and avenasterol. Similarly, storage of rapeseed with an 11–12 % moisture content resulted in high phytosterol losses [17].

There is a very limited number of studies on the stability of phytosterols during storage of oilseeds. Rapeseed oil is rich in phytosterols, and only rice, corn or unconventional oils have higher levels of these components [18, 19]. Moisture content of oilseeds is the critical factor affecting their storability and the extent of changes in endogenous components. It is therefore crucial to protect them and minimize degradation during storage of the oilseeds before processing. To reduce respiration and typical biochemical processes in oilseeds, the moisture content of the seeds has to be reduced to about 8 %, and often drying has to be applied.

The temperatures at which preservation and storage of seeds were conducted, also significantly affects the rate of sterol degradation. In order to prevent an increase in temperature in the grain bulk during storage, seeds need to be successively aired [20]. Rudzińska et al. [14] analyzed changes of phytosterols during drying of rapeseed at 60, 80, 100, 120 °C and near-ambient temperature in laboratory silos. They showed that a higher drying temperature caused a faster degradation of sterols. However, brassicasterol, avenasterol and stigmasterol were affected the most by the applied drying temperature when it was above 80 °C. Dried seeds that were stored at ambient temperature for 12 months also showed sterol degradation. Our results also proved that the storage temperature plays an important role in the degradation of phytosterols in rapeseed.

Gladine et al. [21] modified the process of oil refining in order to reduce the loss of sterols and stated that the quality of seeds seems to be the most important. Their results provide practical information to those who wish to produce rapeseed oil with high levels of phytosterols. It has been proved that losses of phytosterols occur during the drying of oilseeds. In this study it was also proved that unfavorable storage conditions have an equally crucial influence on the degradation of these compounds. It is of particular importance that sterol degradation is closely connected with the formation of the oxidized derivatives, called oxyphytosterols [22], which have cytotoxic properties [23]. Thus as a result of inappropriate storage of rapeseed, not only are valuable biologically active compounds lost, but also conditions are created conducive to the production of toxic substances.

## Conclusions

Rapeseed occupies a leading position in the production of oil crops. Rapeseeds contain nutritionally highly valuable compounds—phytosterols. These compounds at individual stages of vegetable oil production undergo many changes which result in a reduction in their contents in the final product. Losses of phytosterols were observed mainly during the stages of production requiring the application of high temperatures and long-term storage. In order to fully utilize their positive effect, it is necessary to minimize the changes which they may undergo during processing and storage of food. These investigations showed that inappropriate storage conditions of rapeseed may significantly reduce the contents of phytosterols in the raw material and therefore also the final product.
